# Can early change in eating disorder psychopathology predict outcome in guided self-help for binge eating?

**DOI:** 10.1007/s40519-020-01059-3

**Published:** 2020-11-04

**Authors:** Paul E. Jenkins, Lydia Smith, Ceridwen Morgan

**Affiliations:** 1 School of Psychology and Clinical Language Sciences, University of Reading, Reading, RG6 6ES UK; 2 Oxford Health NHS Foundation Trust, Warneford Hospital, Oxford, OX3 7JX UK

**Keywords:** Guided self-help, Receiver operating characteristic, Treatment outcome, Binge eating

## Abstract

**Purpose:**

This study tests the value of a measure of eating disorder (ED) psychopathology in predicting outcome following guided self-help in a non-underweight sample with regular binge eating. It examines whether early reductions in ED psychopathology are associated with remission status at post-treatment.

**Methods:**

Seventy-two adults with bulimia nervosa, binge-eating disorder, or an atypical form of these illnesses received up to ten sessions of cognitive behaviour therapy-based guided self-help. Using a session-by-session measure of eating pathology and associated reliable change indices, response was analysed using receiver operating characteristic analysis to predict outcomes at post-treatment.

**Results:**

In this routine care setting, nearly one-quarter of the sample achieved remission following GSH, approximately two-thirds of whom showed early change in ED psychopathology. Early change prior to session 6 was accurate in predicting later remission. Individuals showing early change did not differ from others on baseline characteristics or rates of attrition.

**Conclusion:**

Data suggest that a majority of those who respond to treatment will do so before the second half of treatment, information that could be used to ensure that evidence-based treatments are used as effectively as possible.

**Level of evidence:**

Level III.

## Introduction

Despite difficulties in accurately predicting outcome following psychological treatments, early symptom change (also known as rapid response) has emerged as a robust predictor across several psychiatric disorders, including eating disorders ([1, 2]; see [3] and [4] for reviews). Early changes in both behavioural symptoms and eating disorder (ED) psychopathology are associated with better outcomes following outpatient treatments based on cognitive behaviour therapy (CBT) [3, 4]. Those who make early change are around twice as likely to achieve remission compared to those who do not [5], a finding demonstrated in intensive outpatient treatment [6], as well as brief [7] and standard [2, 5, 8] CBT for EDs.

Guided self-help (GSH) based on CBT principles is a recommended first-line treatment for bulimia nervosa (BN), binge-eating disorder (BED), and atypical forms of these illnesses not meeting full diagnostic criteria [9, 10]). If GSH is unsuccessful, it is recommended that patients are offered an alternative, such as individual or group CBT. Distinguishing individuals who will benefit from continuation of GSH from those who might be ‘stepped up’ to a more intensive treatment is likely to be more cost-effective, offer wider patient choice, and improve outcomes for a greater number of patients [11, 12].

Studies in this area considering GSH have commonly focused on reduction in binge-eating frequency (typically using ≥ 65% reduction from baseline to the fourth treatment week; [13, 14]) to determine early change, perhaps given that many have focused on single disorders, predominantly BED [13–15]. Secondary analysis from randomised controlled trials has often been used, with less data available from routine care (or ‘real-world’) settings, and therefore may not reflect recommendations that GSH can be used for a variety of EDs (i.e., transdiagnostically).

Varying approaches have been used to study early change [15], resulting in substantial heterogeneity regarding the definition of early improvement and the timing of this change [4]. Assessing change through a measure of ED psychopathology, as opposed to behaviours, has the advantage of being applicable across ED diagnoses [16] as it is not restricted to certain features, such as objective binge eating, which may not be present in all individuals [17]. Further, greater ED psychopathology has been linked to treatment response [2, 5–8], future relapse [18], and is more strongly associated with ED-related impairment than behavioural symptoms [19]. In one study of a transdiagnostic sample of individuals receiving CBT [8], early improvements in psychopathology were associated with post-treatment outcome whereas binge eating and self-induced vomiting were not. Such findings have led to the conclusion that “early change in cognitive symptoms may be a stronger factor than behavioural change in predicting outcome in shorter [CBT-based] treatments” ([7], p. 65). However, no studies have yet looked at the predictive value of ED psychopathology within a sample receiving GSH in routine care.

The aims of the current study are to investigate whether (and the point[s] at which) changes in ED psychopathology predict remission in EDs characterised by recurrent binge eating following GSH treatment. We will compare the baseline characteristics of those who demonstrate change to those who do not. We will also look at a behavioural index of early change, specifically ≥ 65% reduction in binge eating at each week of treatment.

## Method

### Participants

Data were collected from participants (*n* = 77) who began GSH between September 2016 and May 2018 (the final participant ended treatment in June 2018). Each was referred to one of two regional centres for the treatment of EDs, part of the UK National Health Service (NHS), providing treatment to individuals registered with a local physician on a no-fee basis. The services are governed by the same NHS Trust although funding arrangements mean that they cover different geographical areas (based on where the patient’s primary care physician is located). Both were commissioned to provide treatment to adults with a diagnosis of an ED according to ICD-10 [20] or DSM-5 [21] criteria. As such, patient demographics are similar and some staff (see below) work across services.

Diagnoses were established according to DSM-5 criteria [21] by qualified clinicians following clinical interview and agreed upon during multidisciplinary team meetings. Although similar areas are covered by assessing clinicians, the clinical interview is not standardised. Conduct of the study was approved by the Oxford Health NHS Foundation Trust Quality and Audit Department and deemed not to require further ethical approval as it comprised an evaluation of routine practice involving retrospective, routinely collected, de-identified data. Five individuals were excluded from the study as they attended the first session of treatment and then either failed to attend again (*n* = 3) or declined further treatment (*n* = 2).

### Measures

#### Baseline measures

At initial assessment with the service, participants completed:

1. The EDE-Q [22], a self-report measure of eating pathology covering the past 28 days. This questionnaire assesses both cognitive and behavioural aspects of EDs and can produce a total score (EDE-Q Global), which ranges from 0 to 6, with higher scores indicating greater ED psychopathology. The EDE-Q also produces frequency ratings of disordered eating behaviours (e.g., binge eating, self-induced vomiting). The EDE-Q Global has been supported as a reliable measure of symptoms representing the “‘common core’ of psychopathological features underpinning” an ED ([23], p. 201). McDonald’s ω (used to assess internal consistency reliability) was 0.87.
2. The Clinical Impairment Assessment questionnaire (CIA; [24]), a 16-item measure which assesses psychosocial impairment over the past 28 days. Participants rate the degree to which their ED symptoms have impacted aspects of their life (e.g., “made it difficult to concentrate”) on a scale of 0 (not at all) to 3 (a lot). Higher scores (range = 0–48) indicate greater impairment and McDonald’s ω was 0.90. The CIA has been shown to be a useful measure of impairment in similar, transdiagnostic, samples [25].
3. The Clinical Outcomes in Routine Evaluation-Outcome Measure (CORE-OM; [26]), a self-report measure of psychological distress which has been supported for use in ED samples [27]. It comprises 34 items asking participants to rate how frequently they have experienced each item (e.g., “I have felt tense, anxious or nervous”) over the last week. A total score is calculated as a mean of scores on all items (rated from 0 to 4), with higher scores indicating greater distress. McDonald’s *ω* was 0.93.

#### Weekly measurement

As part of treatment, participants were asked to complete the ED-15 [28], a brief self-report measure designed to assess session-by-session change in ED symptoms. Ten items assessing ED psychopathology (e.g., “Been preoccupied with thoughts of food and eating”) are scored on a 0–6 scale reflecting frequency over the past week in addition to five items regarding the frequency of disordered eating behaviours. The instrument’s developers suggest that two subscales (Weight and Shape Concerns; Eating Concerns) can be derived from the ten psychopathology items, in addition to a total score (the mean of these ten items), a model supported in a study of the instrument’s psychometric properties [29]. As correlations between the subscales were high (*r*_s_ = 0.599), the Total score (McDonald’s ω at first session = 0.90) was used to assess psychopathology, similar to the study of Raykos et al. [5] using the EDE-Q Global. The ED-15 Total has been found to correlate strongly with the EDE-Q Global [28, 29], suggesting that they measure similar constructs.

#### Treatment

Participants were provided with a popular self-help book which provides psychoeducation and a CBT approach for the treatment of broadly defined (i.e., addressing both subjective and objective) binge eating [30]. Participants were asked to attend a local clinic, weekly at first, for guidance from one of four trained facilitators. Participants were asked to attend ten face-to-face sessions, provided over 12 weeks. Lasting 20–25 min, sessions occurred weekly at first, with the final two occurring fortnightly. Facilitators comprised junior psychologists working for the NHS who attended weekly supervision from clinical psychologists with experience providing CBT and GSH for EDs. Three facilitators provided treatment in both locations. The ethos behind the guidance is ‘programme-led’, with facilitators supporting patients to make change by offering support and encouragement and keeping a focus on changing eating behaviour [31].

### Reliable and clinically significant change

Jacobson and Truax [32] proposed two metrics to evaluate the impact of psychological therapy. To be considered “recovered”, an individual must show reliable change—where observed change is unlikely to be due to measurement error—and clinically significant change—where scores on a measure move from ‘dysfunctional’ population norms to those of a ‘functional’ population. Reliable change was calculated following the suggested amendment of Christensen and Mendoza [33], whereby change greater than 1.96 times the standard error of the difference is unlikely to have occurred by chance. In the current study, minimum reliable change on the ED-15 Total was 1.34. Regarding calculation of clinically significant change, Tatham et al. [28] provide scores for both a non-clinical and clinical sample: mean (SD) ED-15 Total scores were 2.05 (1.33) and 4.24 (1.09), respectively. Given overlapping distributions of these samples, the midpoint between the two was chosen to represent clinical significance (Jacobson and Truax’s Method *c*; p. 13). The resulting value is 3.254, similar to 1SD above the mean of the non-clinical sample [34].

### Remission classification

Remission was defined by the combination of demonstrating a healthy body mass index (BMI) (≥ 19.0 kg/m^2^), abstinence (no episodes of binge eating, vomiting, or laxative use at the final session according to the ED-15) and both reliable and clinically significant change on the ED-15. This method follows guidance for defining outcome in EDs [35] and adopts a BMI cutoff above which participants are unlikely to experience adverse effects of being ‘underweight’ [36].

### Statistical analyses

Receiver operating characteristic (ROC) analysis was used to determine the point(s) at which change predicted remission at post-treatment. Specifically, the area under the curve (AUC) was used to obtain a probability that an individual who is deemed to have remitted at post-treatment will show change at a given point.

Chi-square or Fisher’s exact test (when sample sizes were small) were used for categorical data and non-parametric (Mann–Whitney *U*) tests for continuous data, given the presence of non-normality. ROC analysis is typically non-parametric and does not assume a normal distribution for the index test; it is therefore particularly well suited to hypothesis testing in routine care settings and less affected by issues such as non-normal distributions [37]. As a secondary aim, we report the timing of attrition—the point at which patients in GSH no longer attended for further sessions. The (non-parametric) Kaplan–Meier method was used to estimate timing of drop-out.

### Sample size estimation

To investigate predictors of change using ROC analysis, at least 66 participants were desired [38], based on a power of 0.8, alpha level of 0.05, and AUC estimates from Nazar et al. [4].

### Missing data

ED-15 Total data were missing completely at random (Little's MCAR, *Χ*^2^ (111) = 109.825, *p* = 0.514). To assess the possible impact of model misspecification [39], multiple imputation (with chained equations) of missing ED-15 Total data was conducted, regardless of why data were missing. Twenty imputed datasets were created, imputed at scale level given high internal consistency [40], with age, diagnosis, completer status, BMI, and baseline ED-15 Total as predictors of missing data, in addition to each subsequent ED-15 Total score. Results did not change the interpretation of data, with changes only in AUC estimates, not significance values. Data are therefore reported without imputation. It was not possible to calculate change indices for one individual who did not provide data for Session Two.

### Timing of symptom change

To investigate predictors of remission at post-treatment, two methods of identifying change in ED psychopathology were tested: patients achieving clinically significant change on the ED-15 Total (i.e., scoring below 3.254) at each session; and patients achieving reliable change (i.e., a reduction in ED-15 Total scores of ≥ 1.34 from the first treatment session). As some patients did not provide data at Session One, we used data from Session Two where this was missing to represent the First Session of treatment, from which change scores were calculated. To investigate behavioural change, individuals were identified who showed ≥ 65% reduction in binge-eating frequency from baseline to each session they attended.

## Results

### Demographic and clinical characteristics

Participants ranged in age from 18 to 79 years (mean = 34.7, SD = 12.8). All had BMIs in the non-underweight range (> 18.5 kg/m^2^) and 93.1% were female. Mean (SD) duration of illness was 17.0 (13.4) years. Based on EDE-Q data at baseline (i.e., previous 28 days), mean (SD) binge eating frequency was 15.81 (10.05) episodes and mean (SD) Global score was 4.32 (0.90). Mean (SD) CIA total was 32.47 (8.43) and 1.95 (0.61) for the CORE-OM.

Of 72 individuals who commenced GSH, 50 completed all ten sessions and a further 4 completed treatment early; thus, attrition was 25.0%. ‘Early completers’ were patients who, following satisfactory response to treatment and progress through the programme, decided with the facilitator that further sessions were not required.^1^ On average, participants received 8.90 (2.15) sessions of support. Twenty-nine individuals (40.3%) reported abstinence at their final session and 22 (30.6%) were classified as “recovered” based on reliable change indices. Sixteen individuals (22.2%) reported both abstinence and recovery and were considered remitted for the purposes of this study. Remission was achieved by 10/38 (26.3%) individuals with BN, 3/12 (25.0%) of those with BED, and 3/22 (13.6%) of those with Other Specified Feeding and Eating Disorder (OSFED).

### Predicting remission

#### Method 1: clinically significant change

As shown in Table 1, an ED-15 score below 3.254 prior to session 8 was significantly associated with remission at post-treatment, as was change prior to Session 10.

**Table 1:** ROC analysis of clinically significant change predicting remission, including numbers achieving change at each session

Session	ED-15 total < 3.254 (*n*)	AUC (95% CI)	Standard error	*p* value
First available (S1 or S2)	13	0.496 (0.286–0.707)	0.107	0.974
3	19	0.557 (0.344–0.770)	0.109	0.596
4	22	0.575 (0.361–0.789)	0.109	0.486
5	21	0.607 (0.396–0.818)	0.108	0.320
6	28	0.604 (0.397–0.810)	0.105	0.336
7	28	0.586 (0.378–0.793)	0.106	0.426
8	23	0.793 (0.623–0.963)	0.087	0.007
9	31	0.668 (0.478–0.857)	0.097	0.119
10	31	0.750 (0.598–0.902)	0.078	0.020

#### Method 2: reliable change

**Fig. 1:**
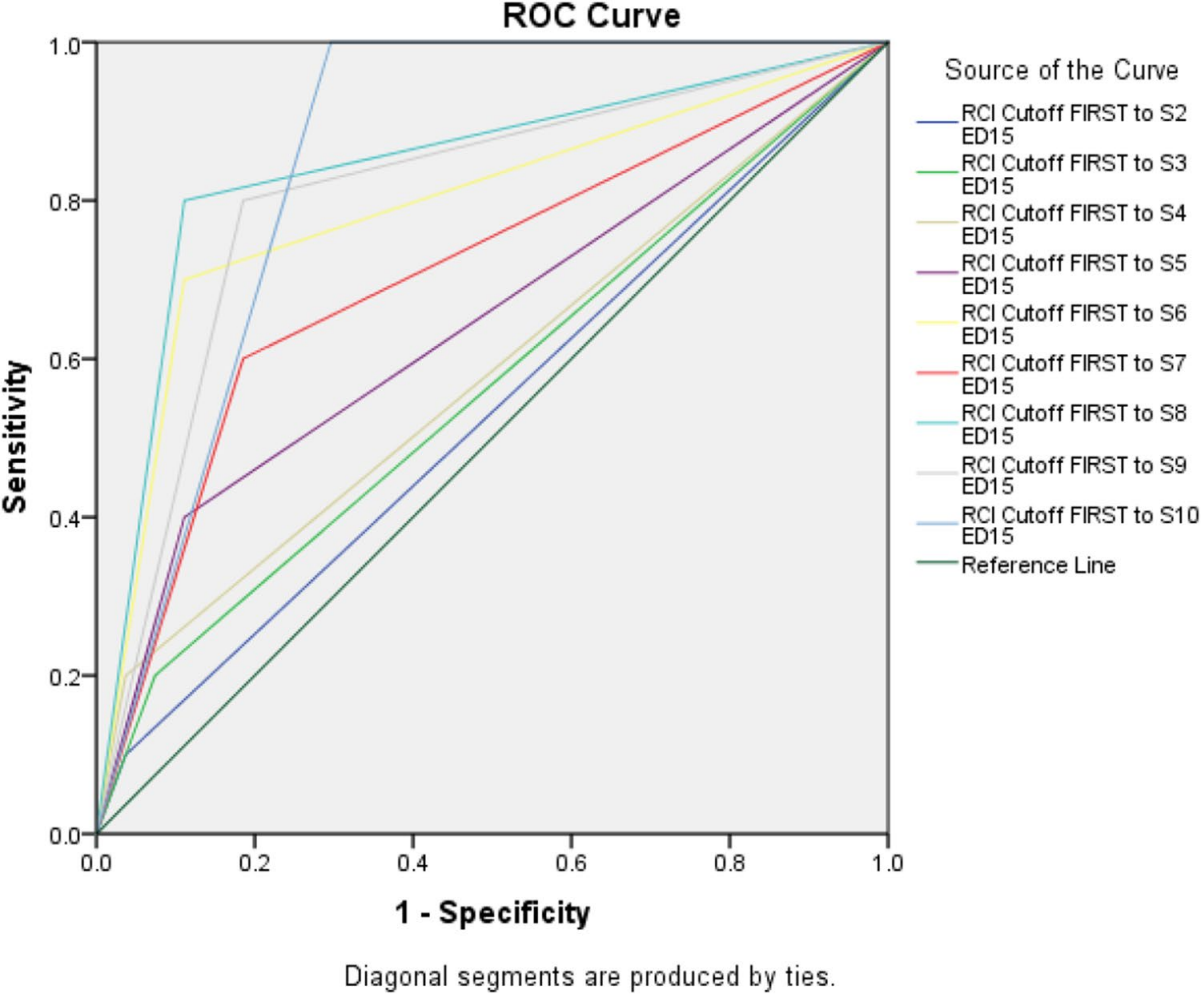
ROC curves for reliable change and remission

A decrease in ED-15 Total of at least 1.34 points before session 6 was associated with post‐treatment remission status (*p* = 0.007; AUC 95% CiIs 0.610–0.979; Table 2). Achieving reliable change at future sessions was associated with continued accuracy, except for change prior to session 7 which only approached significance (*p* = 0.055; 95% CiIs 0.505–0.910). ROC curves are presented in Fig. 1 and suggest that reliable change occurring prior to Session 6 can identify around two-thirds (11/16) of those who achieve remission.

**Table 2:** ROC analysis of reliable change predicting remission (change from first session), including numbers achieving change immediately prior to each session

Session	Reliable change (*n*)	AUC (95% CI)	Standard error	*p* value
2	4	0.531 (0.315–0.748)	0.111	0.771
3	7	0.563 (0.344–0.782)	0.112	0.561
4	5	0.581 (0.360–0.803)	0.113	0.452
5	10	0.644 (0.428–0.861)	0.111	0.182
6	17	0.794 (0.610–0.979)	0.094	0.007
7	20	0.707 (0.505–0.910)	0.103	0.055
8	18	0.844 (0.683–1.000)	0.082	0.001
9	22	0.807 (0.639–0.976)	0.086	0.005
10	24	0.852 (0.732–0.972)	0.061	0.001

#### Method 3: behavioural change

A reduction in frequency of binge eating ≥ 65% from baseline was predictive of remission only prior to Session 6, and this metric otherwise appeared to be a poor predictor of remission (Table 3).

**Table 3:** ROC analysis of reduction in binge eating (≥ 65% from baseline) predicting remission, including numbers achieving change at each session

Session	≥ 65% reduction in binge eating (*n*)	AUC (95% CI)	Standard error	*p* value
2	7	0.558 (0.390–0.726)	0.086	0.481
3	15	0.567 (0.401–0.733)	0.085	0.416
4	21	0.513 (0.351–0.676)	0.083	0.871
5	18	0.500 (0.339–0.661)	0.082	0.999
6	24	0.688 (0.533–0.842)	0.079	0.023
7	26	0.589 (0.428–0.751)	0.083	0.279
8	30	0.594 (0.434–0.753)	0.081	0.255
9	31	0.625 (0.469–0.781)	0.080	0.129
10	30	0.634 (0.478–0.790)	0.080	0.104

### Comparison of groups at baseline

Seventeen individuals achieved reliable change prior to Session 6 (Method 2). There were no significant differences between this group and remaining participants on baseline measures (Table 4).

**Table 4:** Characteristics of groups at baseline (method 2)

Baseline variable	*n*	Mean (SD)		Differences between RC and ARC	
		RC	ARC	Mann–Whitney *U*	*p* value
Age, years	71	35.45 (15.31)	34.55 (11.73)	531.00	0.921
BMI, kg/m^2^	68	29.47 (6.98)	31.08 (9.64)	453.50	0.721
DOI, years	58	16.28 (16.33)	17.38 (12.10)	316.00	0.459
No. of sessions	71	9.64 (0.79)	8.71 (2.30)	463.00	0.241
EDE-Q					
Global	70	4.37 (0.67)	4.26 (0.98)	502.00	0.847
OBEs	68	18.18 (10.29)	14.87 (9.87)	403.00	0.217
Self-induced vomiting	69	14.23 (20.53)	10.11 (18.30)	451.00	0.382
Laxative use	69	1.86 (4.22)	2.70 (5.95)	481.00	0.556
CORE-OM Total	70	1.89 (0.46)	1.96 (0.66)	492.00	0.747
CIA Total	68	33.81 (6.62)	31.67 (9.12)	425.00	0.433

Numbers differ as data were not complete for all participants

*DOI* duration of illness, *ARC* absence of reliable change group, *RC* reliable change group

### Comparison of treatment outcomes

Those showing reliable change did not differ from others on rates of treatment completion (82.4% (14/17) v. 74.1% (40/54); *p* = 0.745, Fisher’s exact test) or number of treatment sessions received (Table 4). Those showing reliable change prior to Session 6 were significantly more likely than others to achieve remission at post-treatment (64.7% (11/17) v. 9.3% (5/54); *χ*^2^ (1) = 22.77, *p* < 0.001, *Φ* = 0.57).

### Attrition

**Fig. 2:**
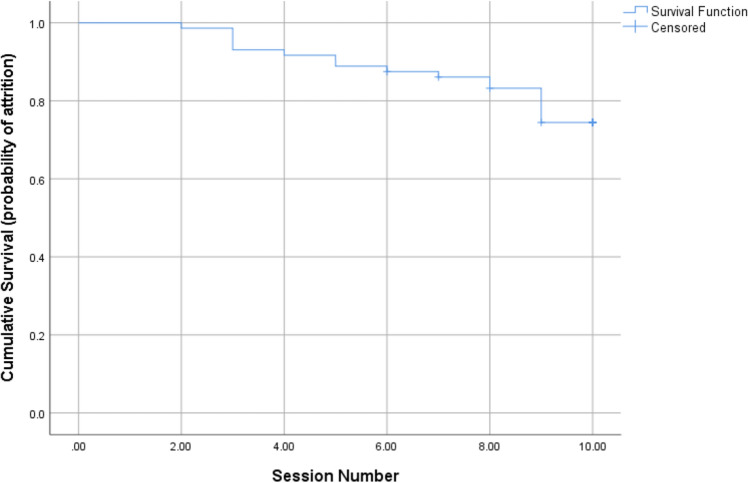
Survival plot (Kaplan–Meier Curve) of timing of dropout during GSH

Figure 2 presents the survival curve, representing the sessions after which patients no longer attended GSH. As noted above, attrition rates were not related to early change status and Mann–Whitney *U* tests suggested no baseline differences between those who dropped out and those who completed treatment (Table 5).

**Table 5:** Characteristics of individuals who dropped out and those who completed treatment

Baseline variable	*n*	Mean (SD)		Group differences	
		Dropped out	Completed	Mann–Whitney *U*	*p* value
Age, years	72	33.56 (9.53)	35.09 (13.76)	486.00	0.999
BMI, kg/m^2^	68	30.08 (10.87)	30.78 (8.29)	380.50	0.453
DOI, years	58	15.69 (8.59)	17.42 (14.57)	286.50	0.911
EDE-Q					
Global	70	4.22 (0.80)	4.35 (0.94)	393.50	0.317
OBEs	68	15.13 (11.32)	16.02 (9.74)	385.00	0.653
Self-induced vomiting	70	7.33 (9.42)	13.10 (21.11)	424.50	0.548
Laxative use	70	5.50 (9.81)	1.85 (3.95)	363.00	0.077
CORE-OM total	70	1.92 (0.69)	1.97 (0.59)	431.50	0.624
CIA total	68	34.29 (8.79)	31.86 (8.30)	345.00	0.210

Numbers differ as data were not complete for all participants

*DOI* duration of illness

## Discussion

The current study extended previous research looking at early symptom change in GSH for EDs characterised by recurrent binge eating. Participants who showed improvements in ED psychopathology before the sixth session of treatment were more likely to achieve remission at post-treatment compared to those who did not. Taken alongside studies of longer forms of CBT for EDs [2, 5], the findings reinforce the view that early response remains “the only consistent indicator of optimal prognosis across treatments” ([5], p. 767).

The current study, which extends analysis of early change to a transdiagnostic sample of individuals receiving GSH, found no baseline differences between rapid responders and others. Rates of attrition (at around 25%) were similar across groups. As previous authors have noted, those who demonstrate early change are not merely “easy” cases who can be identified according to clinical severity ([13], p. 388), a conclusion partially supported by the high levels of symptom and impairment noted in this sample.

The findings could have potential to expediently identify individuals who respond well to low-intensity treatments for binge eating and thus inform stepped care models for ED treatment [9, 10]. Use of a measure of ED psychopathology suggested that around one-quarter of patients who start treatment can be accurately classed as rapid responders [5], although this proportion is lower than in other studies using behavioural criteria [14, 15]. Assessing early change in such a way (as opposed to a behavioural index) can be implemented across samples of mixed ED groups [5, 12] to predict treatment response and may be particularly useful in routine care situations where mixed diagnostic groups present for treatment. Further, in the current study, behavioural change was a poor predictor of outcome, although it is notable that the only significant association which emerged was also prior to Session 6, suggesting that there is an association between early cognitive and behavioural change [8].

Rates of attrition seen in the current study are similar to those from systematic reviews [41]. Whilst numbers were small, the present findings suggest that attrition occurs over the course of treatment, although a large number failed to attend the final session (i.e., after Session 9, which was the modal number). Given the limited data available, it is difficult to form conclusions but this could reflect avoidance towards the end of treatment or that participants had made sufficient gains that they did not feel a final session was necessary [42]. Similar patterns have been observed in group-based CBT for atypical EDs [43] although other literature suggests that attrition tends to occur earlier in treatment [44] and reviews in this area have noted the limited data on the timing of dropout [41].

Identifying how best to help individuals who do not show early change should remain a research priority. More intensive treatment is an option, and several CBT-based interventions for EDs consider ‘therapeutic termination’ of treatment if patients appear unable to commit to goals around change [45, 46]. The current study suggests that limited change prior to Session 6 of GSH could be used to prompt such a discussion, and the findings might inform further research looking at patients who benefit from a ‘therapeutic termination’ and those who may require more intensive treatment.

Some limitations of the study are notable. Although a comprehensive definition of remission was used, including both behavioural and cognitive symptoms, the timeframe was short and limited by self-report, thus not representing the “gold standard” of assessing outcome in such designs [47]. Overlaps in the measures used may have provided artefactual correlation towards the end of treatment, although findings regarding early change are in line with previous work [3]. The study was also limited by recruitment from one NHS Trust and provided GSH with ten sessions of support, more than some GSH studies [15], which may have influenced the findings. The sample was intended to represent a transdiagnostic sample characterised by regular binge eating (broadly defined), but small numbers limit generalisation across the range of EDs, particularly OSFED. Given the paucity of validated session-by-session measures in EDs, further studies are required to confirm the psychometric properties of the ED-15 and similar measures [28] and to replicate the findings presented here.

In summary, the current study offers support for use of an easily computed measure of symptom change in GSH that may be useful in informing stepped-care models [9, 10]. Monitoring ED psychopathology throughout treatment can help clinicians focus on helping patients achieve early change [2] and consider tailored approaches for those who are likely to respond more slowly [3, 5]. Conducting a formal review after around 4 weeks of treatment has been recommended for this purpose [7].

## What is already known on this subject?

Early behavioural change has been consistently associated with positive outcomes following treatment for EDs. However, early change in ED psychopathology has been given less attention with limited exploration of this variable in guided self-help treatment.

## What this study adds

This study extends findings regarding the predictive ability of early change in ED treatment to include a guided self-help approach. Change in ED psychopathology prior to Session 6 of treatment accurately predicted remission in a transdiagnostic sample. The study has implications for stepped care models of treatment, including early (non-)response.

## Footnotes

1 These individuals ended treatment after 6, 7, 8, and 9 sessions respectively.
